# Supernatant from *Bifidobacterium* Differentially Modulates Transduction Signaling Pathways for Biological Functions of Human Dendritic Cells

**DOI:** 10.1371/journal.pone.0002753

**Published:** 2008-07-23

**Authors:** Cyrille Hoarau, Laurence Martin, Delphine Faugaret, Christophe Baron, Audrey Dauba, Cécile Aubert-Jacquin, Florence Velge-Roussel, Yvon Lebranchu

**Affiliations:** 1 UPRES EA 4245 « Cellules Dendritiques & Greffes », Université François-Rabelais, Tours, France; 2 Département de Recherche et Développement Blédina, Villefranche sur Saône, France; 3 UFR des Sciences Pharmaceutiques, Université François-Rabelais, Tours, France; University of Sheffield, United Kingdom

## Abstract

**Background:**

Probiotic bacteria have been shown to modulate immune responses and could have therapeutic effects in allergic and inflammatory disorders. However, the signaling pathways engaged by probiotics are poorly understood. We have previously reported that a fermentation product from *Bifidobacterium breve* C50 (BbC50sn) could induce maturation, high IL-10 production and prolonged survival of DCs via a TLR2 pathway. We therefore studied the roles of mitogen-activated protein kinases (MAPK), glycogen synthase kinase-3 (GSK3) and phosphatidylinositol 3-kinase (PI3K) pathways on biological functions of human monocyte-derived DCs treated with BbC50sn.

**Methodology/Principal Findings:**

DCs were differentiated from human monocytes with IL-4 and GM-CSF for 5 days and cultured with BbC50sn, lipopolysaccharide (LPS) or Zymosan, with or without specific inhibitors of p38MAPK (SB203580), ERK (PD98059), PI3K (LY294002) and GSK3 (SB216763). We found that 1) the PI3K pathway was positively involved in the prolonged DC survival induced by BbC50sn, LPS and Zymosan in contrast to p38MAPK and GSK3 which negatively regulated DC survival; 2) p38MAPK and PI3K were positively involved in DC maturation, in contrast to ERK and GSK3 which negatively regulated DC maturation; 3) ERK and PI3K were positively involved in DC-IL-10 production, in contrast to GSK3 that was positively involved in DC-IL-12 production whereas p38MAPK was positively involved in both; 4) BbC50sn induced a PI3K/Akt phosphorylation similar to Zymosan and a p38MAPK phosphorylation similar to LPS.

**Conclusion/Significance:**

We report for the first time that a fermentation product of a bifidobacteria can differentially activate MAPK, GSK3 and PI3K in order to modulate DC biological functions. These results give new insights on the fine-tuned balance between the maintenance of normal mucosal homeostasis to commensal and probiotic bacteria and the specific inflammatory immune responses to pathogen bacteria.

## Introduction

The functional ability of dendritic cells (DCs) to generate specific immune responses depends on the levels of costimulatory molecule expression, cytokine production profile and survival of DCs [1], [2]. These properties result from the integration of different intracellular signals induced by the microenvironment, particularly exposure to bacteria [3]. The immune system differentiates commensal bacteria (resulting in no inflammatory responses) and pathogen bacteria (resulting in inflammatory responses). One of the mechanisms involved could be the integration of the differential signaling induced by pathogen-recognition receptors (PRRs). Toll-Like Receptors (TLRs) are PRRs expressed on DCs which recognize pathogen-associated molecular patterns (PAMPs) from bacteria corresponding to a broad spectrum of highly conserved microbial structures [4]. TLRs are members of the IL-1 receptor (IL-1-R) superfamily characterized by an intracytoplasmic Toll-IL-1 receptor (TIR) domain, which mediates recruitment of the interleukin-1 receptor-associated kinase (IRAK) complex and downstream signaling, via adapter molecules such as MyD88 [4]. It was initially suggested that signaling through any of the TLRs instructs DCs to promote Th1 responses [5]. However, TLR engagement can induce a wide variety of signal transduction pathways to regulate the nature, magnitude and duration of immune responses [2], [6], [7]. Probiotic bacteria have been shown to modulate immune responses, particularly mucosal immunity, and could have therapeutic effects in allergic and inflammatory disorders [8]–[10]. In particular, probiotic bacteria can interact with monocyte-derived DCs to modulate their properties [11], [12]. However, the signaling pathways engaged by probiotics are poorly understood, particularly the ways that differ from the inflammatory signaling pathways induced by pathogenic bacteria [13]–[19]. We have previously reported that a fermentation product from *Bifidobacterium breve* (BbC50sn) could induce maturation, high IL-10 production and prolonged survival of DCs via a TLR2 pathway [20]. Nuclear factor-kappa B (NF-κB) activation was involved in the maturation process of DCs treated by BbC50sn (BbC50sn-DCs). However, IL-10 production and prolonged DC survival were independent of NF-κB, suggesting other intracellular pathways induced by BbC50sn. Interestingly, BbC50sn was able to suppress the biological effects of lipopolysaccharide (LPS) on IL-12 production and DC apoptosis, confirming that different signaling pathways are involved in DC biology. Moreover, if NF-κB activation is required for DC maturation after TLR engagement, other intracellular pathways, such as mitogen-activated protein kinases (MAPK), glycogen synthase kinase-3 (GSK3) and phosphatidylinositol 3-kinase (PI3K) pathways, seem to be critical in the biological functions of DCs [21]–[26]. We therefore studied the roles of these kinases in the regulation of activation, maturation and survival induced by BbC50sn on human monocyte-derived DCs using specific inhibitors.

## Results

### Survival of BbC50sn, LPS and Zymosan-stimulated DC was enhanced by PI3K, with an opposite effect of p38MAPK and GSK3 signaling pathways

**Figure 1:**
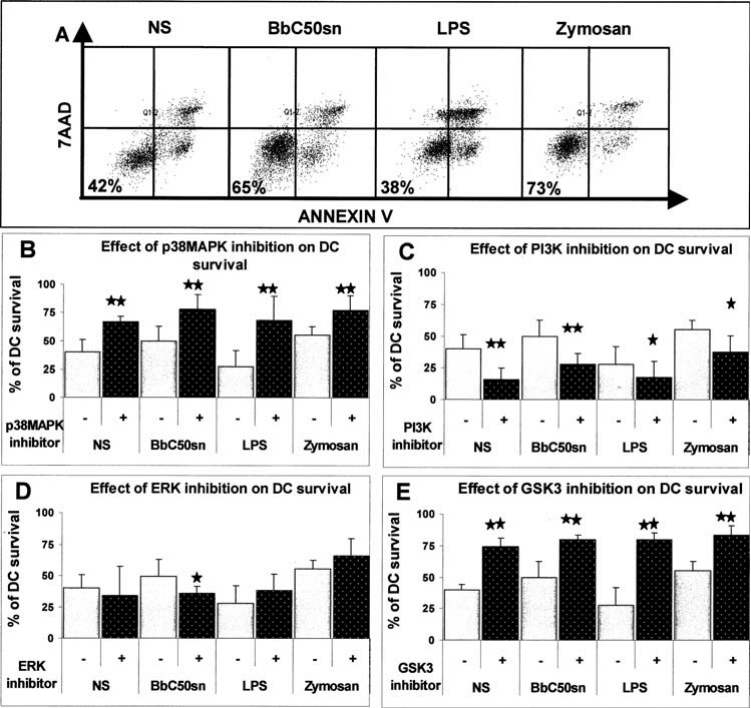
BbC50sn-DC survival with the PI3K, p38MAPK, ERK and GSK3 inhibitors. DCs were used either unstimulated (NS) or in the presence of BbC50sn (100 µg/ml), LPS (50 ng/ml) or Zymosan (25 µg/ml) for 8 days. The results are expressed as: A) the percentage of cell survival (double Annexin V and 7AAD-negative cells) without inhibitors; B, C, D, E) the percentage of cell survival as mean±SD of 5 different experiments B) with and without SB203580 (20 µM), C) with and without PD98059 (25 µM), D) with and without LY294002 (10 µM) E) with and without SB216763 (10 µM). The effects of kinase inhibitors were compared for panel B, C, D and E with the same control data for each stimulation. Statistical analysis was performed using the Wilcoxon test for paired non-parametric data. Significance is indicated by p value * p≤0.05; **p≤0.001.

As previously described [20], BbC50sn induced prolonged DC survival compared to LPS after 8 days of stimulation. Zymosan, a TLR-2 agonist, induced a DC survival similar to that induced by BbC50sn (Fig. 1A). In order to study the involvement of signaling pathways in BbC50sn-DC survival, we added specific kinase inhibitors to the culture medium 1 hour before the addition of the different TLR agonists. The dosage of kinase inhibitors was chosen in order to avoid a non toxicity (data not shown). The p38MAPK inhibitor (SB203580; 20 µM) increased DC survival, whatever the TLR agonist: BbC50sn (50%±14 vs 78%±14; p = 0.008, n = 5), LPS (28%±14 vs 69%±22, p = 0.008, n = 4), Zymosan (56%±8 vs 77%±13, p = 0.008, n = 5) (Fig. 1B). Interestingly the DC survival observed with LPS after addition of the p38MAPK inhibitor was similar to that observed with BbC50sn. In contrast, the PI3K inhibitor (LY294002; 10 µM) reduced BbC50sn-DC survival (50%±14 vs 27%±9, p = 0.004, n = 4) (Fig. 1C), and also LPS-DC (28%±14 vs 18%±11, p = 0.031, n = 5) and Zymosan-DC (56%±8 vs 37%±12, p = 0.02, n = 5) survival, with a dose-dependent effect (data not shown). The ERK inhibitor (PD98059; 25 µM) did not significantly change DC survival after LPS or Zymosan stimulation, but reduced survival after for BbC50sn stimulation (50%±14 vs 35%±5, p = 0.048, n = 5) (Fig. 1D). The GSK3 inhibitor (SB216763; 10 µM) increased survival of BbC50sn-DC (50%±14 VS 85%±6, p<0.001, n = 4); LPS-DC (28%±14 vs 80%±7, p<0.001, n = 4) and Zymosan-DC (56%±8 vs 88%±6, p<0.001, n = 4) (Fig. 1E). PI3K activation therefore induces DC survival whereas p38MAPK and GSK3 reduce DC survival. The differences observed in survival between BbC50sn-DC and LPS-DC could be due to preferential activation of PI3K by BbC50sn and Zymosan and preferential activation of p38MAPK by LPS.

### Maturation of BbC50sn, LPS and Zymosan-stimulated DC was enhanced by p38MAPK and PI3K, with opposite effects of ERK and GSK3 signaling pathways

**Figure 2:**
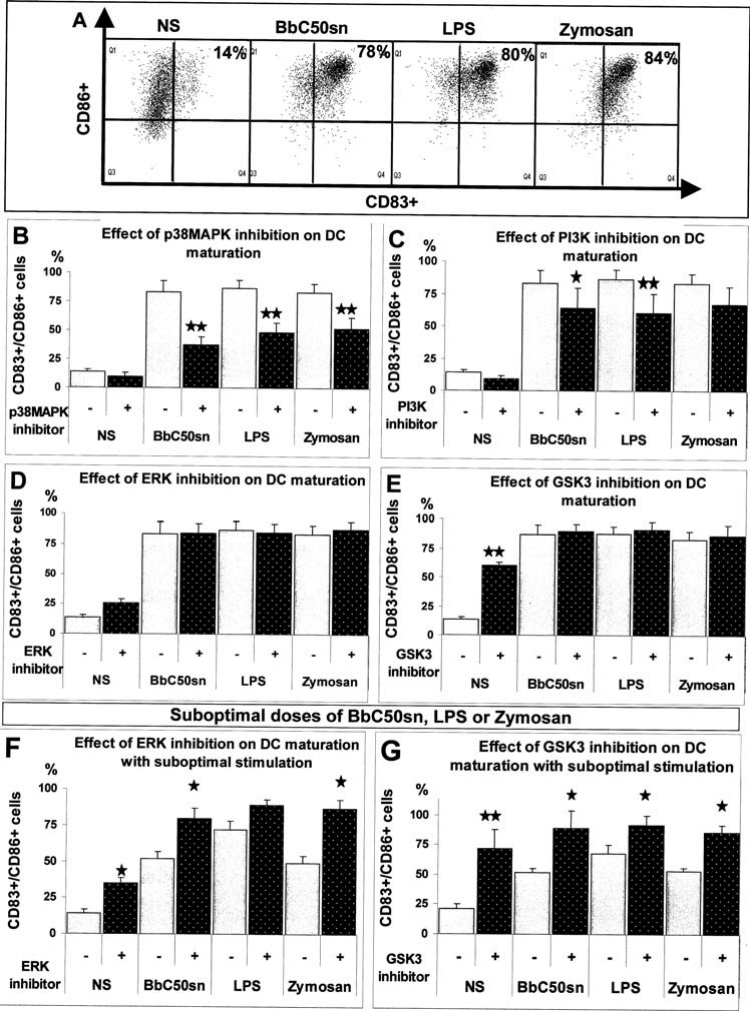
BbC50sn-DC maturation with the PI3K, p38MAPK, ERK and GSK3 inhibitors. In panel A, B, C, D and E, DCs were used either unstimulated (NS) or in the presence of BbC50sn (100 µg/ml), LPS (50 ng/ml) or Zymosan (25 µg/ml) for 2 days. The results are expressed as A) the percentage of CD83 and CD86 double positive cells without inhibitors; B, C, D, E) the percentage of CD83 and CD86 double positive cells as mean±SD of 6 different experiments, B) with and without SB203580, C) with and without PD98059, D) with and without LY294002, E) with and without SB216763 with the same inhibitors doses as in Fig 1. The effects of kinase inhibitors in panels B, C, D and E were compared with the same control data for each stimulation. In panels F and G, DCs were used either unstimulated (NS) or in the presence of sub-optimal doses of BbC50sn (10 µg/ml), LPS (5 ng/ml) or Zymosan (2.5 µg/ml) for 2 days, with the same inhibitors that in panels 2D and 2E respectively. The results are expressed as the percentage of CD83 and CD86 double positive cells F) with and without PD98059, G) with and without SB216763, with the same inhibitors doses as in Fig 1. The effects of kinase inhibitors in panels F and G were compared with the same control data for each stimulation. Statistical analysis was performed using the Wilcoxon test for paired non-parametric data. Significance is indicated by p value :* p≤0.05; **p≤0.001.

BbC50sn induced DC maturation, with up-regulation of CD83 and CD86 in the same proportions as did LPS and Zymosan 2 days after stimulation (Fig. 2A). The p38MAPK inhibitor (SB203580) induced a profound reduction in CD83 and CD86 DC expression after BbC50sn (83%±10 vs 38%±7, p<0.001, n = 6), LPS (87%±7 vs 48%±8, p<0.001, n = 6) and Zymosan (83%±7 vs 52%±9, p<0.001, n = 6) stimulation (Fig. 2B). The PI3K inhibitor (LY294002) induced a reduction in CD83 and CD86 DC expression after BbC50sn (83%±10 vs 64%±15, p = 0.05, p = 4), LPS (87%±7 vs 60%±14, p<0.001, n = 4) and Zymosan (83%±7 vs 67%±13, p = 0.06, n = 4) stimulation (Fig. 2C). In contrast, the ERK (PD98059) and GSK3 (SB216763) inhibitors did not modify DC maturation after BbC50sn, LPS or Zymosan stimulation, but increased maturation of unstimulated cells (Fig. 2D; 2E). Furthermore, when sub-optimal doses of BbC50sn, LPS or Zymosan (10 µg/ml, 5 ng/ml, 5 µg/ml, respectively) were used, the addition of ERK and GSK3 inhibitors induced full DC maturation (Fig. 2F; 2G). p38MAPK and to a lesser extent PI3K are therefore both positively involved in DC maturation whereas ERK and GSK3 activation inhibit DC maturation.

### Cytokine production of BbC50sn, LPS and Zymosan-stimulated DC was differentially regulated by p38MAPK, ERK, GSK3 and PI3K signaling pathways

**Figure 3:**
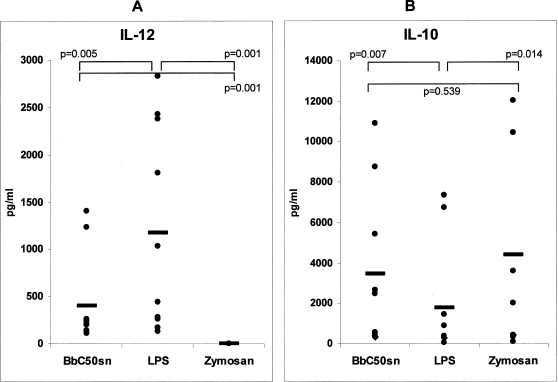
BbC50sn-DC cytokine production. DCs were stimulated with either BbC50sn (100 µg/ml) or LPS (50 ng/ml) or Zymosan (25 µg/ml) for 2 days. IL-12 (A) or IL-10 (B). Synthesis were analyzed by ELISA in the supernatant of these cultures after 48 h of stimulation. The results are expressed in picograms per milliliter: each point represents the values of one donor for each stimulation and the heavy bar the mean of cytokine production for each stimulus. Statistical analysis was performed using the Wilcoxon test for paired non-parametric data. Significance is indicated by p value.

**Figure 4:**
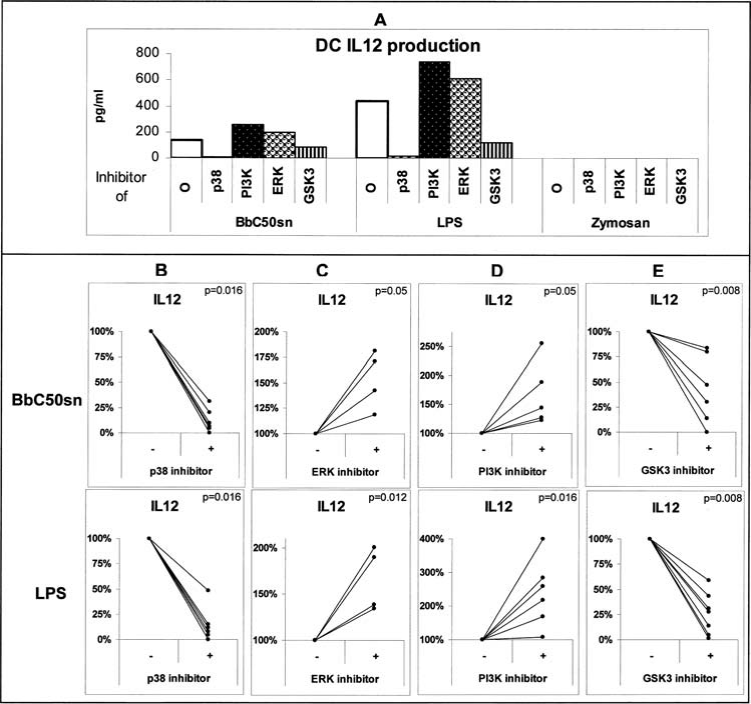
BbC50sn-DC IL12 production with the PI3K, p38MAPK, ERK and GSK3 inhibitors. DCs were stimulated by BbC50sn (100 µg/ml) or LPS (50 ng/ml) for 2 days. A) with and without SB203580; one representative experiment, B) with and without PD98059, C) with and without LY294002, D) with and without SB216763 with the same inhibitor doses as in Fig 1. The results, determined by ELISA, are expressed in percentage of the levels measured for each donor with and without inhibitors and represent 4 to 7 different experiments. Statistical analysis was performed using the Wilcoxon test for paired non-parametric data. Significance is indicated by p value.

**Figure 5:**
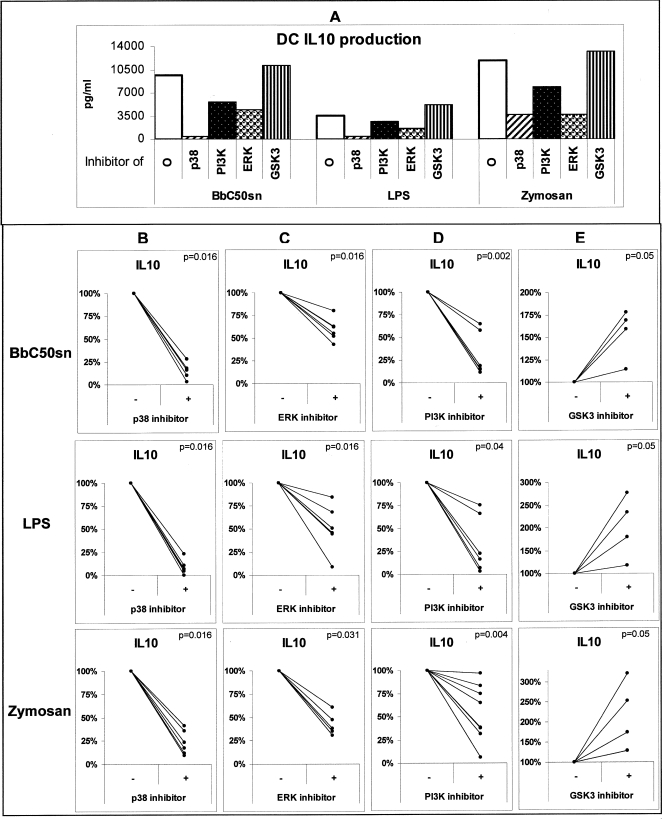
BbC50sn-DC IL10 production with the PI3K, p38MAPK, ERK and GSK3 inhibitors. DCs were used in the same conditions as in Fig 3: in the presence of either BbC50sn or LPS or Zymosan for 2 days: A) with and without SB203580 one representative experiment, B) with and without PD98059, C) with and without LY294002, D) with and without SB216763 with the same inhibitor doses as in Fig 1. IL-10 production in culture supernatants was determined by ELISA. The results are expressed in percentage of the levels measured for each donor with and without inhibitors of 4 to 7 different experiments. Statistical analysis was performed using the Wilcoxon test for paired non-parametric data. Significance is indicated by p value.

Monocyte-derived DCs were activated by BbC50sn, LPS or Zymosan and cytokine synthesis was analysed by ELISA in the supernatant of these cultures. BbC50sn-treated DCs produced low IL-12 and high IL-10 levels in contrast to LPS-treated DCs (404 pg/ml±480 vs 1175 pg/ml±1070, p = 0.005, n = 10 (Fig. 3A) and 3444 pg/ml±3700 vs 1780 pg/ml±2800, p = 0.007, n = 10 (Fig. 3B), respectively). No significant IL-12 production was measurable after Zymosan stimulation (Fig. 3A), whereas IL-10 production was high (3175 pg/ml±4400 (Fig. 3B)). Due to the high variability of cytokine productions between donors (Fig. 3), we chose to express the results in percentage of the cytokine levels measured in the absence of kinase inhibitors to analyse their effects on IL-12 (Fig. 4) and IL-10 (Fig. 5) DC production. The p38MAPK inhibitor (SB203580) induced a near total reduction in IL-12 production after BbC50sn and LPS stimulation (Fig. 4B). The GSK3 inhibitor (SB216367) also induced a reduction in IL-12 production after BbC50sn and LPS stimulation (Fig. 4E). In contrast, the ERK inhibitor (PD98059) and the PI3K inhibitor (LY294002) induced increases in IL-12 DC production after BbC50sn or LPS stimulation (Fig. 4CD). Zymosan was unable to produce measurable IL-12 levels, even in the presence of ERK and PI3K inhibitors (Fig. 4A).

The p38MAPK inhibitor (SB203580), and to a lesser extent the ERK inhibitor (PD98059) and the PI3K inhibitor (LY294002), induced a significant reduction in IL-10 production by DCs after BbC50sn, Zymosan or LPS stimulation (Fig. 5BCD). In contrast, the GSK3 inhibitor (SB216367) induced an increase in IL-10 production after BbC50sn, LPS or Zymosan (Fig. 5E). These results suggest that p38MAPK is positively involved in both IL-12 and IL-10 DC production, that PI3K and ERK decrease IL-12 and increase IL-10 production and that GSK3 decreases IL-10 and increases IL-12 production.

**Figure 6:**
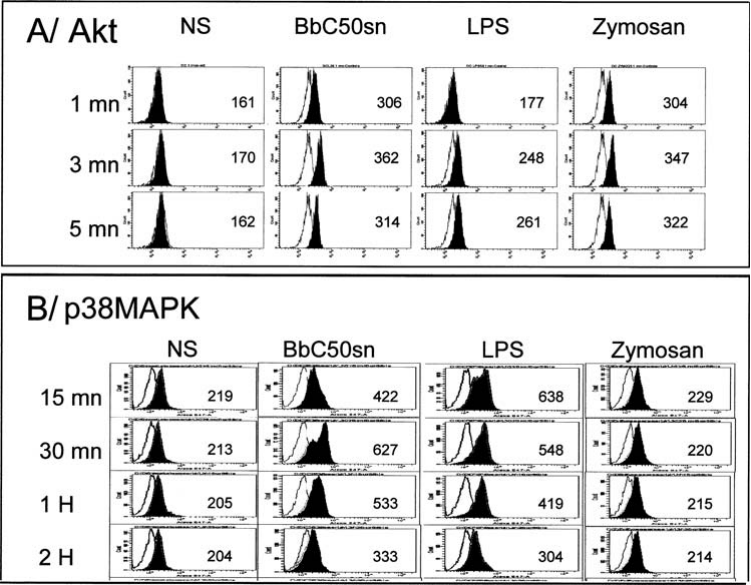
BbC50sn induced both Akt and p38MAPK phosphorylations. Akt and p38MAPK phosphorylations induced by either BbC50sn or LPS or Zymosan in DC were evaluated at different times by flow cytometry and expressed in mean fluorescence intensity (MFI) for A) Akt; B) p38MAPK. Black histograms represent staining of phosphorylated kinases and empty or gray histograms correspond to isotype controls for Akt and p38MAPK respectively. These results are representative of one out of 2 experiments.

The differences in cytokine production observed between BbC50sn- and LPS-DC (Fig. 4A, 5A) could be due to a preferential activation of PI3K by BbC50sn as in DC survival. But the differences observed between BbC50sn and Zymosan (Fig. 4A, 5A) suggest that BbC50sn is also able to activate p38MAPK although to a lesser extent. We therefore studied the phosphorylation of Akt and p38MAPK in DC after stimulation by BbC50sn, LPS or Zymosan. As shown in Fig. 6, BbC50sn induced a phosphorylation of Akt similar to that observed with Zymosan, but a phosphorylation of p38MAPK similar to that observed with LPS.

## Discussion

We have previously observed that NF-κB activation was involved in the maturation process of DCs stimulated by a supernatant of a *Bifidobacterium* fermentation product [20]. But in contrast to Menard who reported anti-inflammatory properties of a similar Bifidobacteria strain with decreased NFκB nuclear translocation [27], our results, concerning a product of fermentation and not the supernatant of the bacteria alone, didn't show modification of IL-10 production and prolonged DC survival after NF-κB inhibition by lactacystin, suggesting the involvement of other intracellular pathways. In the present study, we demonstrated for the first time that BbC50sn induces maturation, activation and survival of dendritic cells via different signaling pathways. We used kinase inhibitors mostly used in the literature in order to compare our results with other publications. Moreover, these inhibitors, SB203580, LY294002, PD98059, and SB216763 seem to be the most specific inhibitors described for respectively p38MAPK, PI3K, ERK and GSK3 [28]. We found that 1) the PI3K pathway is positively involved in the prolonged DC survival induced by BbC50sn whereas p38MAPK and GSK3 have negative effects; 2) p38MAPK and PI3K are both positively involved in DC maturation, in contrast to ERK and GSK3; 3) PI3K and ERK are positively involved in DC-IL-10 production, in contrast to GSK3 that is positively involved in DC-IL-12 production and p38MAPK that is positively involved in both. 4) BbC50sn induced a PI3K/Akt phosphorylation similar to that induced by Zymosan and a p38MAPK phosphorylation similar to that induced by LPS. Furthermore, the preferential involvement of some of these different pathways after BbC50sn, LPS and Zymosan stimulation could explain the different properties observed with these agonists.

We observed that the PI3K pathway was involved in the prolonged DC survival measured after 8 days of stimulation by BbC50sn. PI3K has recently been shown to be directly involved in TLR signaling pathways, independently of the IRAK/TRAF6/NF-κB pathway [29], [30]. PI3K therefore constitutes a good candidate for DC signaling pathway after the TLR2 engagement by BbC50sn or Zymosan. Few studies have reported the involvement of PI3K in DC survival. Xie *et al* reported that PI3K was essential for DC survival during the differentiation of monocytes in immature DCs, independently of any TLR stimulation [31]. Ardeshna *et al* demonstrated that PI3K was involved in myeloid DC survival by modulating the balance of pro- and anti-apoptotic Bcl-2/Bad family proteins which could be induced by BbC50sn, as we have previously described [20], [32]. Interestingly, the same authors found that p38MAPK induced DC survival measured 48 h after LPS stimulation, in contrast to our results where p38MAPK decreased long term DC survival measured on day 8 after BbC50sn, LPS and Zymosan stimulation. This suggests that p38MAPK may have a positive effect on early DC survival and a negative effect on long term DC survival. We also showed for the first time that inhibition of GSK3 increased DC survival. GSK3 is a serine protein kinase involved in maturation, activation and apoptosis of several cells [33]–[35]. It has been reported that PI3K neutralizes GSK3 activity, via Akt phosphorylation [33]. Therefore, the prolonged DC survival that we observed with BbC50sn could be partly the consequence of GSK3 inhibition induced by PI3K and Akt.

We also found that p38MAPK was involved in the maturation of DCs treated by BbC50sn. This is in accordance with previous studies on monocyte-derived DCs stimulated with LPS or Zymosan [32], [36]–[38]. The p38MAPK signaling pathway positively regulates DC maturation, with increased CD80, CD86, CD83 and HLA-DR expression [39]. In contrast, we observed that ERK and GSK3 inhibitors increased DC maturation after suboptimal stimulation with either BbC50sn, LPS or Zymosan, which confirmed that p38MAPK and ERK have opposite effects on DC maturation [25], [40], [41]. We also observed that inhibition of PI3K reduced DC maturation after BbC50sn, LPS and Zymosan stimulation but at lower levels than with the p38MAPK inhibitor. This suggests that PI3K is positively involved in DC maturation. This could be due in part to the inhibition of GSK3 induced by PI3K and Akt, because we observed that GSK3 inhibitor increased DC maturation. Our results are in accordance with those of Rodionova *et al* who recently reported that GSK3 inhibits spontaneous DC maturation and that GSK3 activity was inhibited by Akt after TLR engagement during the maturation process [35]. In many cases, inhibition of p38MAPK and to a lesser extent PI3K have the same effect on DC maturation whether microbial or non-microbial stimuli as CD40L trimers are used [42]. However, ours results suggested that the intensity of the kinase recruitment is different between LPS or BbC50sn and Zymosan (Fig. 2 and Fig. 6).

In terms of cytokine production, the p38MAPK *and GSK3* inhibitors decreased DC IL-12 production after BbC50sn and LPS stimulation, in contrast to PI3K and ERK inhibitors which increased IL-12 production. These results are in accordance with those of Agrawal *et al* who reported that p38MAPK is positively involved in DC IL-12 production after LPS stimulation of human monocyte-derived DC [25]. They observed that the magnitude and kinetics of MAPK phosphorylation depended on the TLR agonist involved: TLR4 activation induced a positive p38MAPK/ERK ratio in contrast to TLR2. p38MAPK positively regulates IL-12 production after TLR4 engagement and ERK negatively regulates IL-12 production after TLR2 engagement. As for maturation, p38MAPK and ERK have opposite effects on DC IL-12 production [36], [37], [39], [40], [43]. Furthermore, we found that GSK3 inhibitor decreased DC IL-12 production after BbC50sn and LPS stimulation, which is in accordance with the literature concerning monocytes and DCs [34], 35. Moreover, we observed that the PI3K inhibitor increased IL-12 production, which could be related to a regulatory function of this kinase in DC cytokine production through inhibition of GSK3 [44]. Martin *et al* reported that PI3K also induced ERK phosphorylation on human monocytes after TLR2 engagement [45]. The increased DC IL-12 production observed with the PI3K inhibitor in our study could therefore also be the consequence of a reduction in ERK phosphorylation.

**Figure 7:**
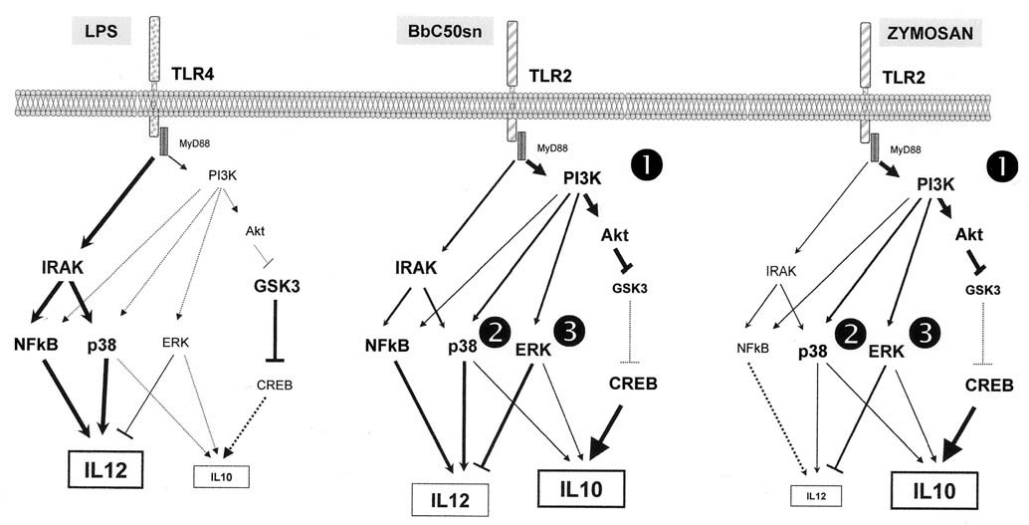
Scheme of putative signaling pathways involved in cytokine production induced by BbC50sn, LPS or Zymosan in DCs. PI3K decreased IL-12 and increased IL-10 production with BbC50sn and Zymosan through (1) phosphorylation of Akt which inhibits GSK3 (inhibitor of CREB = IL-10 nuclear factor); (2) phosphorylation of ERK which positively regulates IL-10 in contrast to IL-12 production; (3) phosphorylation of p38MAPK which positively regulates IL-10 and IL-12 productions. Putative main pathways are represented in bold.

We observed that ERK, p38MAPK and PI3K inhibitors decreased IL-10 production. This is in accordance with several studies which reported the role of ERK in DC IL-10 production [37], [39], [40], [43]. Although, ERK and p38MAPK had opposite effects on IL-12 production, we observed that p38MAPK inhibitor also reduced DC IL-10 production after BbC50sn, LPS and Zymosan stimulation. Foey *et al* also reported that p38MAPK is involved in monocyte IL-10 production [46] and Messmer *et al* found the same effect of p38MAPK on DC IL-10 production [47]. p38MAPK could be involved in the stability of cytokine mRNA at a post-transcriptional level, and this could explain the reduction in both IL-12 and IL-10 production [48]. Because BbC50sn and Zymosan stimulations, in comparison with LPS, result in a higher level of IL10 production and a greater Akt phosphorylation, we hypothesize that PI3K/Akt had a key role in the control of the IL-10/IL-12 DC production balance (Fig. 7). This action could be mediated by GSK3 activity which negatively regulates cAMP response element-binding (CREB), previously described as an IL-10 transcription factor [49]. In conclusion, few studies have investigated the DC intracellular pathways induced by probiotic bacteria [19]. In previous study [20], we have demonstrated that the BbC50sn activity on maturation of DC was dependant of the media in which BbC50 had grown. Indeed, we did saw DC activation by BbC50sn when BbC50 had been cultured in media containing hydrolyzed whey protein as protein source, but not in other media in which dairy proteins contents were different (data not shown). In addition, contrary to BbC50sn the supernatant of BbC7 obtained by the fermentation of BbC7 in media containing hydrolyzed whey protein was not able to trigger DC maturation. Thus the effect of BbC50sn on DC is both media and strain-dependant. Regarding this conclusion, we hypothesis that the nature of the compound(s) could be : metabolite(s) produced during the fermentation ; after dialysis, glycoproteins are the main chemical compounds produced during fermentation and/or bacterial fragments of which composition is modified by the composition of the fermentation media. In this study, we report for the first time that a fermentation product of a bifidobacteria can differentially activate MAPK, GSK3 and PI3K in order to modulate the maturation, activation and survival of DCs to promote a regulatory profile. We observed that PI3K is positively involved in the effetc of BbC50sn on 1) the prolonged DC survival; 2) the maturation; 3) the balance of IL-10/IL-12 production. Nevertheless, the DC p38MAPK phosphorylation induced by BbC50sn could explain some of the properties observed with this fermentation product. Description of the differential modulation of the intracellular signaling induced by PAMPs is important to understand the fine-tuned balance between the maintenance of normal mucosal homeostasis to commensal and fermentation products of bacteria and the specific inflammatory immune responses to pathogen bacteria. Therefore, a better knowledge of the molecular mechanisms of signaling pathways induced by probiotic bacteria, could allow new therapeutic strategies of allergic and autoimmune diseases.

## Methods

### Medium, cytokines, monoclonal antibodies, kinase inhibitors and reagents of cell culture

The culture medium used was RPMI 1640 (Gibco, Cergy Pontoise, France) supplemented with 50 IU/mL penicillin, 50 IU/mL streptomycin (Gibco), 2 mM L-glutamin (Gibco) and 10% heat-inactivated fetal calf serum (FCS) (Gibco). Recombinant human IL-4 was obtained from R&D Systems (Abingdon, United Kingdom), GM-CSF from AbcysSA (Paris, France), LPS from Sigma-Aldrich (St Quentin Fallavier, France) and Zymosan from Invivogen (Toulouse, France). Kinase inhibitors, SB203580 which directly inhibits p38MAPK activity [28], [49], PD98059 which prevents activation of MAPK kinase (MEK) upstream activators of MAPK 3 and 1 (ERK), [50] and SB216763 which prevents activation of GSK3 were obtained from Sigma-Aldrich (St Quentin Fallavier, France), and LY294002 which inhibits PI3K (prevents Akt phosphorylation) from Cell Signaling. The following mouse anti-human mAbs were used for cytometry analysis: fluorescein isothiocyanate (FITC)-anti-CD83 (IgG1, HB15e) and phycoerythrin (PE)-anti-CD86 (IgG2b, HA5), purchased from Immunotech (Marseille, France). Signaling protein phosphorylations were analyzed by FACS with monoclonal antibodies specific for phosphorylated forms of Akt, p38MAPK and ERK (PE coupled for Akt, Alexa 647 for p38MAPK and ERK, from Becton Dickinson, Rungis France). Control cells were stained with corresponding isotype-matched control mAbs (Immunotech and Becton Dickinson). (FITC)-labeled Annexin V (5 µL/1×10^5^ cells) and 7-amino actinomycin D (7-AAD, 10 µg/mL) were used for apoptosis analysis (Becton Dickinson, Rungis, France; Sigma, St Quentin Fallavier, France).

### Production of *Bifidobacterium breve* supernatant

Bifidobacteria were isolated from infant stools as *Bifidobacterium breve* and the strain was called C50 (BbC50). BbC50 was cultured in the presence of hydrolyzed cow's whey. Fermentation was carried out at 37°C under anaerobic conditions for 15 hours. The supernatant of the culture medium was collected by high speed centrifugation after fermentation and concentrated by ultrafiltration (300 kDa), and then dialyzed on a 10 kDa membrane. After concentration, the supernatant was lyophilized for use and called BbC50sn. All the results reported here were obtained with the same batch. BbC50sn activity was evaluated by its ability to promote both increase in bifidobacteria and reduction of *Clostridium* and *Bacteroides* pullulation in mouse gut [51].

### Differentiation and maturation of dendritic cells

Blood of healthy volunteer donors was obtained from cytapheresis after informed consent. Human peripheral blood mononuclear cells (PBMC) were then isolated over Ficoll hypaque and 2×10^8^ were plated in a 175 cm^2^ flask in complete culture medium. After 45 min at 37°C, nonadherent cells were discarded and adherent cells were cultured in the presence of 25 ng/mL recombinant human IL-4 and 1000 IU/mL GM-CSF. After 5 days, 15 to 20×10^6^ cells were harvested, washed and resuspended in culture medium with IL-4 and GM-CSF. DC purity was 97.3%+/−1.4 SD (determined according to CD1a positive cells by flow cytometry). Kinase inhibitors, when mentioned, were added 1 hour before BbC50sn (100 µg/ml), LPS (50 ng/ml) or Zymozan (25 µg/ml). DCs were harvested after stimulation, washed and used for cytometry analysis or functional assays.

### Analysis of cell surface molecules, measurement of apoptosis and protein phosphorylation analysis by flow cytometry

Monocyte-derived dendritic cells were harvested and 1 to 2×10^5^ cells/sample were resuspended in phosphate-buffered saline (PBS). For maturation analysis, cells were then incubated with saturating concentrations of the different fluorochrome-conjugated monoclonal antibodies for 30 min at 4°C. The stained cells were washed twice in PBS and fixed in 0.5% paraformaldehyde PBS solution until analysis by flow cytometry. Cell surface expression was then analyzed using a laser flow cytometer (FACSCanto®, BD, Mountain View, USA). Data were analyzed for the percentage of marker-positive cells (at least 10,000 cells/sample were analyzed using Diva® software (Becton Dickinson).

For measurement of apoptosis, DCs were incubated with Annexin V and 7-AAD. The proportions of positive and negative 7-amino actinomycin D (7-AAD) and fluorescein isothiocyanate (FITC)-labeled Annexin V cells were determined by flow cytometry (FACSCanto®, Becton Dickinson). Double Annexin V and 7AAD-negative cells corresponded to cell survival.

Signaling protein phosphorylations were studied by FACS. Briefly, DC were incubated at 37°C with either BbC50sn or Zymosan or LPS, then fixed and permeabilized using a commercially available cell permeabilization reagent kit (Caltag Laboaratories). Finally, cells were stained with specific Abs for 1 hour at room temperature, then washed once in PBS-2% human albumin serum (HAS) and resuspended in PBS until analysis by flow cytometry.

#### Cytokine quantification in culture supernatant

Measurements of IL-12 (p70) and IL-10 levels were performed by human enzyme-linked immunosorbent assay (ELISA) using commercially available antibodies and standards according to the manufacturer's protocols (eBioscience).

#### Statistical analysis

Results are expressed as the mean±standard deviation (SD) unless otherwise stated. Comparison between samples with and without kinase inhibitors was conducted using the Wilcoxon test for paired non-parametric data. Analyses were performed using XLSTAT 2008 Software V2.03. A value of p<0.05 was considered as significant.

## References

[1] Kalinski P, Smits HH, Schuitemaker JH, Vieira PL, van Eijk M (2000). IL-4 is a mediator of IL-12p70 induction by human Th2 cells: reversal of polarized Th2 phenotype by dendritic cells.. J Immunol.

[2] Pulendran B, Kumar P, Cutler CW, Mohamadzadeh M, Van Dyke T (2001). Lipopolysaccharides from distinct pathogens induce different classes of immune responses in vivo.. J Immunol.

[3] Kapsenberg ML (2003). Dendritic-cell control of pathogen-driven T-cell polarization.. Nat Rev Immunol.

[4] Akira S (2003). Mammalian Toll-like receptors.. Curr Opin Immunol.

[5] Medzhitov R, Janeway C (2000). Innate immune recognition: mechanisms and pathways.. Immunol Rev.

[6] Dinarello CA (2000). Proinflammatory cytokines.. Chest.

[7] MacDonald AS, Straw AD, Bauman B, Pearce EJ (2001). CD8- dendritic cell activation status plays an integral role in influencing Th2 response development.. J Immunol.

[8] Kalliomaki M, Salminen S, Poussa T, Arvilommi H, Isolauri E (2003). Probiotics and prevention of atopic disease: 4-year follow-up of a randomised placebo-controlled trial.. Lancet.

[9] Ukena SN, Singh A, Dringenberg U, Engelhardt R, Seidler U (2007). Probiotic Escherichia coli Nissle 1917 Inhibits Leaky Gut by Enhancing Mucosal Integrity.. PLoS ONE.

[10] Moro G, Arslanoglu S, Boehm G (2007). Reducing the burden of atopic dermatitis–authors' response.. Arch Dis Child.

[11] Smits HH, Engering A, van der Kleij D, de Jong EC, Schipper K (2005). Selective probiotic bacteria induce IL-10-producing regulatory T cells in vitro by modulating dendritic cell function through dendritic cell-specific intercellular adhesion molecule 3-grabbing nonintegrin.. J Allergy Clin Immunol.

[12] Foligne B, Zoumpopoulou G, Dewulf J, Ben Younes A, Chareyre F (2007). A key role of dendritic cells in probiotic functionality.. PLoS ONE.

[13] Christensen HR, Frokiaer H, Pestka JJ (2002). Lactobacilli differentially modulate expression of cytokines and maturation surface markers in murine dendritic cells.. J Immunol.

[14] Braat H, de Jong EC, van den Brande JM, Kapsenberg ML, Peppelenbosch MP (2004). Dichotomy between Lactobacillus rhamnosus and Klebsiella pneumoniae on dendritic cell phenotype and function.. J Mol Med.

[15] Drakes M, Blanchard T, Czinn S (2004). Bacterial probiotic modulation of dendritic cells.. Infect Immun.

[16] Braat H, van den Brande J, van Tol E, Hommes D, Peppelenbosch M (2004). Lactobacillus rhamnosus induces peripheral hyporesponsiveness in stimulated CD4+ T cells via modulation of dendritic cell function.. Am J Clin Nutr.

[17] Strobel S, Mowat AM (2006). Oral tolerance and allergic responses to food proteins.. Curr Opin Allergy Clin Immunol.

[18] O'Hara AM, O'Regan P, Fanning A, O'Mahony C, Macsharry J (2006). Functional modulation of human intestinal epithelial cell responses by Bifidobacterium infantis and Lactobacillus salivarius.. Immunology.

[19] Kim YG, Ohta T, Takahashi T, Kushiro A, Nomoto K (2006). Probiotic Lactobacillus casei activates innate immunity via NF-kappaB and p38 MAP kinase signaling pathways.. Microbes Infect.

[20] Hoarau C, Lagaraine C, Martin L, Velge-Roussel F, Lebranchu Y (2006). Supernatant of Bifidobacterium breve induces dendritic cell maturation, activation, and survival through a Toll-like receptor 2 pathway.. J Allergy Clin Immunol.

[21] Medzhitov R (2001). Toll-like receptors and innate immunity.. Nat Rev Immunol.

[22] Takeda K, Kaisho T, Akira S (2003). Toll-like receptors.. Annu Rev Immunol.

[23] Arbibe L, Mira JP, Teusch N, Kline L, Guha M (2000). Toll-like receptor 2-mediated NF-kappa B activation requires a Rac1-dependent pathway.. Nat Immunol.

[24] Loscher CE, Draper E, Leavy O, Kelleher D, Mills KH (2005). Conjugated linoleic acid suppresses NF-kappa B activation and IL-12 production in dendritic cells through ERK-mediated IL-10 induction.. J Immunol.

[25] Agrawal S, Agrawal A, Doughty B, Gerwitz A, Blenis J (2003). Cutting edge: different Toll-like receptor agonists instruct dendritic cells to induce distinct Th responses via differential modulation of extracellular signal-regulated kinase-mitogen-activated protein kinase and c-Fos.. J Immunol.

[26] Dillon S, Agrawal A, Van Dyke T, Landreth G, McCauley L (2004). A Toll-like receptor 2 ligand stimulates Th2 responses in vivo, via induction of extracellular signal-regulated kinase mitogen-activated protein kinase and c-Fos in dendritic cells.. J Immunol.

[27] Menard S, Candalh C, Bambou JC, Terpend K, Cerf-Bensussan N (2004). Lactic acid bacteria secrete metabolites retaining anti-inflammatory properties after intestinal transport.. Gut.

[28] Davies SP, Reddy H, Caivano M, Cohen P (2000). Specificity and mechanism of action of some commonly used protein kinase inhibitors.. Biochem J.

[29] Strassheim D, Asehnoune K, Park JS, Kim JY, He Q (2004). Phosphoinositide 3-kinase and Akt occupy central roles in inflammatory responses of Toll-like receptor 2-stimulated neutrophils.. J Immunol.

[30] Hoarau C, Gerard B, Lescanne E, Henry D, Francois S (2007). TLR9 activation induces normal neutrophil responses in a child with IRAK-4 deficiency: involvement of the direct PI3K pathway.. J Immunol.

[31] Xie J, Qian J, Yang J, Wang S, Freeman ME, 3rd (2005). Critical roles of Raf/MEK/ERK and PI3K/AKT signaling and inactivation of p38 MAP kinase in the differentiation and survival of monocyte-derived immature dendritic cells.. Exp Hematol.

[32] Ardeshna KM, Pizzey AR, Devereux S, Khwaja A (2000). The PI3 kinase, p38 SAP kinase, and NF-kappaB signal transduction pathways are involved in the survival and maturation of lipopolysaccharide-stimulated human monocyte-derived dendritic cells.. Blood.

[33] Jope RS, Johnson GV (2004). The glamour and gloom of glycogen synthase kinase-3.. Trends Biochem Sci.

[34] Martin M, Rehani K, Jope RS, Michalek SM (2005). Toll-like receptor-mediated cytokine production is differentially regulated by glycogen synthase kinase 3.. Nat Immunol.

[35] Rodionova E, Conzelmann M, Maraskovsky E, Hess M, Kirsch M (2007). GSK-3 mediates differentiation and activation of proinflammatory dendritic cells.. Blood.

[36] Nakahara T, Uchi H, Urabe K, Chen Q, Furue M (2004). Role of c-Jun N-terminal kinase on lipopolysaccharide induced maturation of human monocyte-derived dendritic cells.. Int Immunol.

[37] Dillon S, Agrawal S, Banerjee K, Letterio J, Denning TL (2006). Yeast zymosan, a stimulus for TLR2 and dectin-1, induces regulatory antigen-presenting cells and immunological tolerance.. J Clin Invest.

[38] Chang L, Karin M (2001). Mammalian MAP kinase signalling cascades.. Nature.

[39] Arrighi JF, Rebsamen M, Rousset F, Kindler V, Hauser C (2001). A critical role for p38 mitogen-activated protein kinase in the maturation of human blood-derived dendritic cells induced by lipopolysaccharide, TNF-alpha, and contact sensitizers.. J Immunol.

[40] Puig-Kroger A, Relloso M, Fernandez-Capetillo O, Zubiaga A, Silva A (2001). Extracellular signal-regulated protein kinase signaling pathway negatively regulates the phenotypic and functional maturation of monocyte-derived human dendritic cells.. Blood.

[41] Aiba S, Manome H, Nakagawa S, Mollah ZU, Mizuashi M (2003). p38 Mitogen-activated protein kinase and extracellular signal-regulated kinases play distinct roles in the activation of dendritic cells by two representative haptens, NiCl2 and 2,4-dinitrochlorobenzene.. J Invest Dermatol.

[42] Yu Q, Kovacs C, Yue FY, Ostrowski MA (2004). The role of the p38 mitogen-activated protein kinase, extracellular signal-regulated kinase, and phosphoinositide-3-OH kinase signal transduction pathways in CD40 ligand-induced dendritic cell activation and expansion of virus-specific CD8+ T cell memory responses.. J Immunol.

[43] Dillon TJ, Karpitski V, Wetzel SA, Parker DC, Shaw AS (2003). Ectopic B-Raf expression enhances extracellular signal-regulated kinase (ERK) signaling in T cells and prevents antigen-presenting cell-induced anergy.. J Biol Chem.

[44] Fukao T, Koyasu S (2003). PI3K and negative regulation of TLR signaling.. Trends Immunol.

[45] Martin M, Schifferle RE, Cuesta N, Vogel SN, Katz J (2003). Role of the phosphatidylinositol 3 kinase-Akt pathway in the regulation of IL-10 and IL-12 by Porphyromonas gingivalis lipopolysaccharide.. J Immunol.

[46] Foey AD, Parry SL, Williams LM, Feldmann M, Foxwell BM (1998). Regulation of monocyte IL-10 synthesis by endogenous IL-1 and TNF-alpha: role of the p38 and p42/44 mitogen-activated protein kinases.. J Immunol.

[47] Messmer D, Hatsukari I, Hitosugi N, Schmidt-Wolf IG, Singhal PC (2006). Morphine reciprocally regulates IL-10 and IL-12 production by monocyte-derived human dendritic cells and enhances T cell activation.. Mol Med.

[48] Brook M, Sully G, Clark AR, Saklatvala J (2000). Regulation of tumour necrosis factor alpha mRNA stability by the mitogen-activated protein kinase p38 signalling cascade.. FEBS Lett.

[49] Tong L, Pav S, White DM, Rogers S, Crane KM (1997). A highly specific inhibitor of human p38 MAP kinase binds in the ATP pocket.. Nat Struct Biol.

[50] Dudley DT, Pang L, Decker SJ, Bridges AJ, Saltiel AR (1995). A synthetic inhibitor of the mitogen-activated protein kinase cascade.. Proc Natl Acad Sci U S A.

[51] Lievin V, Peiffer I, Hudault S, Rochat F, Brassart D (2000). Bifidobacterium strains from resident infant human gastrointestinal microflora exert antimicrobial activity.. Gut.

